# Allelopathic inhibition of the extracts of *Landoltia punctata* on *Microcystis aeruginosa*

**DOI:** 10.1080/15592324.2022.2058256

**Published:** 2022-04-05

**Authors:** Li Dan, Li Peng, Yan Zhiqiang, Li Na, Yao Lunguang, Cao Lingling

**Affiliations:** a Collaborative Innovation Center of Water Security for Water Source Region of Mid-route Project of South-North Water Diversion of Henan Province, Nanyang Normal University, Henan, China; b College of Life Sciences and Agricultural Engineering, Nanyang Normal University, Nanyang, Henan, China

**Keywords:** *Landoltia punctata*, *Microcystis aeruginosa*, ethyl acetate extract, allelopathy, mechanism

## Abstract

To study the allelopathic effect of the extracts of *Landoltia punctata*, the changes of cell density of *Microcystis aeruginosa* were measured. The anti-algae allelopathic effect of different organic solvent extracts of *L. punctata* was evaluated, and the physiological, biochemical indexes were determined to discuss the mechanism of algal inhibition. The results showed that the petroleum ether, dichloromethane and ethyl acetate extracts showed various inhibitory effects on *M. aeruginosa*. Among them, ethyl acetate extract was the most strongly allelopathic part with the semi-effect concentration(EC_50_) of 59.6 mg L^−1^, the central polarity part of inhibitory activity. The contents of chlorophyll a(Chl a) and phycobiliproteins(PBPs) of *M. aeruginosa* were decreased under the concentration of 200 mg L^−1^ ethyl acetate extract, which indicated that the photosynthesis of *M. aeruginosa* was inhibited. The consent of microcystins was lower compared to control under 200 mg L^−1^. The contents of superoxide dismutase(SOD), malondialdehyde(MDA) and hydrogen peroxide(H_2_O_2_) of cell pellets were firstly increased and then decreased, which suggested that the algal cells were seriously damaged by oxidation. The results indicated that the extracts of *L. punctata* had inhibitory effect on *M. aeruginosa*, and the ethyl acetate extract was the central part of the inhibitory substances, which affected photosynthesis and caused peroxidation damage to inhibit cell proliferation. These findings will be helpful for exploration and application of allelopathic effects of *L. punctata* in harmful algae control.

## 1 Introduction

In recent years, cyanobacteria bloom caused by eutrophication have attracted more and more attention by researchers.^1^ *Microcystis aeruginosa* is the most widely distributed cyanobacteria, which can produce toxins and pollute water. The toxins could reduce the photosynthetic capacity of other plants and cause damage through the biological chain into the animals directly or indirectly.^2,3^ Therefore, it is an urgent issue to prevent and control *M. aeruginosa* for water environmental protection at present.

Various strategies have been reported to control algal blooms, including physical, chemical and biological methods. The physical method, such as ultrasound techniques, UV irradiation, membrane filtration technology and adsorption, is simple to operate, but it takes a long time and consumes a large of human and financial resources.^4^ The chemical method, such as use photosensitizers, herbicides and other chemicals, is often used for emergency treatment, but it often causes secondary pollution to water and ecological risk.^4,5^ Compared with chemical and physical methods, natural plant extracts, due to their effective inhibition, low cost, natural availability, no secondary pollution and other advantages, have received much attention.^6^ Allelopathy is defined as any direct or indirect beneficial or harmful effects of an organism on others via the release of chemical substances, known as allelochemicals, into the environment.^6^ Over the years, exploiting the allelopathy of hydrophytes which can release chemical compounds into the water to inhibit the growth of algae has attracted much attention.^7^ Allelopathy method to inhibit the growth of algae is less pollution and the allelochemicals are easy degradation under natural conditions.^8^ Therefore, it has significant environmental and ecological significance to control algae with allelopathy.

Numerous plants, especially aquatic plants, have been reported for their allelopathic effect.^9^ For example, *Eichhornia crassipes, Sagittaria trifolia, Ceratophyllum demersum* and *Lemna minor* L can release allelochemicals and compete for nutrients to control the growth of algae.^10–13^ *Landoltia punctata* is a floating herbaceous plant, which can grow fast and possessed efficient Cu (II), Mn (II) and Zn (II)ions adsorption effect and was used in sewage treatment.^14^ It is reported that the cultured water of *L. punctata* had an allelopathic effect on *M. aeruginosa* which the concentration was 2 × 10^8^ cell mL^−1^ under co-culture and significantly reduced in 1.6 × 10^9^ cells/L for *M. aeruginosa*.^15,16^ However, the effects of allelochemicals released by *L. punctata* on algae growth have not been reported. In this study, the extracts of *L. punctata* were investigated on the growth and the inhibitory mechanism of ethyl acetate extract against *M. aeruginosa* was studied, which provide a application of *L. punctata* in eutrophic water remediation.

## 2 Materials and methods

### 2.1 Materials

The strain of *M. aeruginosa* (FACHB-977) was purchased from the freshwater algae species bank of the Institute of Hydrobiology, CAS (Wuhan, China). The *M. aeruginosa* was cultivated in BG-11 medium at 25 ± 1°C under a cool white fluorescent light of 3000 lx on a light-dark period 14:10 h(L:D) in an illuminated incubator (GZP-250, Jinghong, Shanghai, China).^17^

*L. punctata* was purchased from the Institute of Hydrobiology, CAS (Wuhan, China), using the hydroponic to expand cultivating. *L. punctata* samples of 2 Kg were dried in an oven at 40°C to a constant weight and crushed into power.

### 2.2 *Preparation of extracts of* L. punctata

The power of *L. punctata* was extracted with 95% ethanol, the ratio of sample to solvent was 1:10 (m/v) for 24 h and extracted three times. The extract was concentrated with rotary evaporator (RV3, IKA, Germany) to obtain the ethanol extract of *L. punctata*.

The ethanol extract was dissolved with five times volume of warm water about 40°C, and extracted with three solvents of petroleum ether, dichloromethane and ethyl acetate at room temperature respectively.^2^ Each solvent was extracted three times, and the solvent liquid was combined and concentrated to obtain petroleum ether, dichloromethane, ethyl acetate and aqueous phase extracts.

### 2.3 Determination of growth density

Algal cell density was calculated by measuring the optical density of algal solution with an ultraviolet-visible luminometer (TU-1810) at 680 nm wavelength.^18^ The initial concentration of the algae solution was 2.7 × 10^6^ cell mL^−1^ with the absorption value was 0.15 at 680 nm. The crude extracts of 100 mg was dissolved in the solvent of 250 μL dimethyl sulfoxide (DMSO), and filtrated with 0.22 μm organic system membrane. The extracts were added to the *M. aeruginosa* culture medium (100 mL) with the final concentration of 200 mg L^−1^ separately. The same volume of DMSO solution was added to the control group with three replicates. The absorption value at 680 nm of the algae solution was recorded every 24 h.

Among the result of preliminary screening, the ethyl acetate extract was selected for follow-up testing. A series of ethyl acetate extract were added into the algae solution and the final concentrations were 12.5, 25, 50, 100 and 200 mg L^−1^, respectively. Three replicates are set for each concentration. The absorption value of the algae solution was recorded every two days. The inhibition rate (IR) was according to the formula.^2^

(1) $$IR=\left[1-\left(En/Cn\right)\right]\times 100\%$$

where En and Cn represent the absorption value at 680 nm of experimental and control groups, respectively.

### 2.4 Determination of chlorophyll a

The Chl a of *M. aeruginosa* was extracted and examined at 649 and 665 nm using an ultraviolet-visible luminometer, and calculated based on the method raised by Lichtenthaler and Wellburn.^19,20^ Algal solution of 10 mL was taken every 48 h, centrifugation at 5000 r min^−1^ for 10 min. cell pellets was added 2 mL of 95% ethanol and put at 4°C without light. Then, the solution was centrifuged at 5000 r min^−1^ for 10 min, and the supernatant was taken for absorption value. The Chl a was calculated by the following equations.

(2) $$Chl\;a\left(mg\;L^{-1}\right)=13.35OD_{665}-6.88OD_{649}$$

### 2.5 Determination of phycobiliproteins

The phycobiliproteins (PBPs) included phycocyanin (PC), allophycocyanin (APC) and phycoerythrin (PE). The algae solution of 5 mL was collected and centrifugated at 4°C, then resuspended in 5 mL of phosphate-buffered solution (PBS) (50 mM, pH 7.28). The resuspension was frozen at −80°C for 8 h, and thawed in the dark at room temperature. The freezing-thawing cycle was repeated three times. The absorbance of supernatant of lysate was spectrophotometrically detected at wavelengths of 650, 620 and 565 nm, respectively. The PC, APC and PE contents were calculated by the following equations.^21^

(3) $$PC\left(mg\;L^{-1}\right)=\left(OD_{620}-0.7*OD_{650}\right)/7.38$$

(4) $$APC\left(mg\;L^{-1}\right)=\left(OD_{650}-0.19*OD_{620}\right)/5.65$$

(5) $$PE\left(mg\;L^{-1}\right)=\left(OD_{565}-2.8*PC-1.34*APC\right)/1.27$$

### 2.6 Determination of H_2_O_2_, antioxidant response and MDA content

To analyze dynamics of H_2_O_2_, superoxide dismutase and MDA contents, 10 mL of culture was sampled every 24 h during test, respectively. Microcystis cells collected by centrifugation were re-suspended in 1 mL of PBS and were thoroughly crushed by freezing-thawing cycles for thrice. The supernatant was taken to detect antioxidases SOD, H_2_O_2_ and MDA content by specific kits which were purchased from the Jiancheng Bioengineering Institute (Nanjing, China), and following specification.

### 2.7 Determination of microcystins

Culture samples of 10 mL were taken every 48 h, centrifugation at 7000 r min^−1^ for 10 min at 4°C. The precipitate was washed twice with distilled water, then centrifuged at 7000 r min^−1^ for 10 min at 4°C. The precipitate was added 2 mL of distilled water and heated in the boiled water for 15 min and broken ultrasonic for 10 min on ultrasonic cleaner (KQ5200DE, Kunshan Shumei, China). The mixture was centrifuged at 12000 r min^−1^ for 10 min at 4°C. The liquid supernatant was extracted with carbon tetrachloride and the water phase was collected for HPLC (LC-20A, Shimadzu, Japan).^22,23^ The microcystins (MC-LR) has a specific absorption peak at 238 nm. The retention time of the sample was compared with that of the MC-LR standard sample (purity of ≥95%), which was purchased from Shanghai Maclin Biochemical Technology Co., LTD (Shanghai, China). According to the peak area at this time, the content of MC-LR can be calculated. The recovery rate for the analysis of MC-LR was 95.0 ± 1.5%.

### 2.8 Statistical analysis

All assays were determined in triplicate, and all data are presented as the mean ± standard error of the mean, one-way ANOVA was used to compare the difference between control group and experimental group using the statistical analysis software of Statistical Package for Social Science 17. When p < .05, results were defined as significant. The semi-effect concentration EC_50_ was calculated by GaphaPad Prism7.0.

## 3 Results

### 3.1 *Effect of* L. punctata *extracts on the growth of* M. aeruginosa

**Figure 1.**
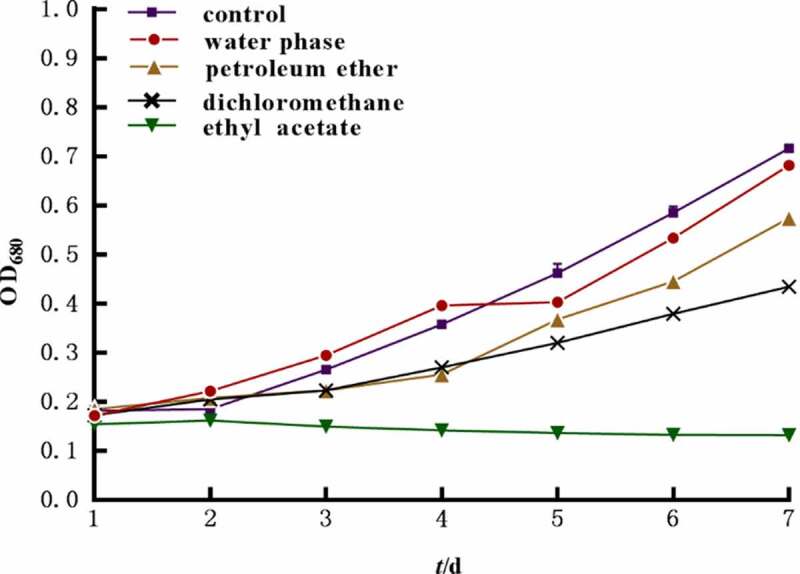
Absorption value at 680 nm of different polarity parts of *L. punctata* on *M. aeruginosa.*

**Figure 2.**
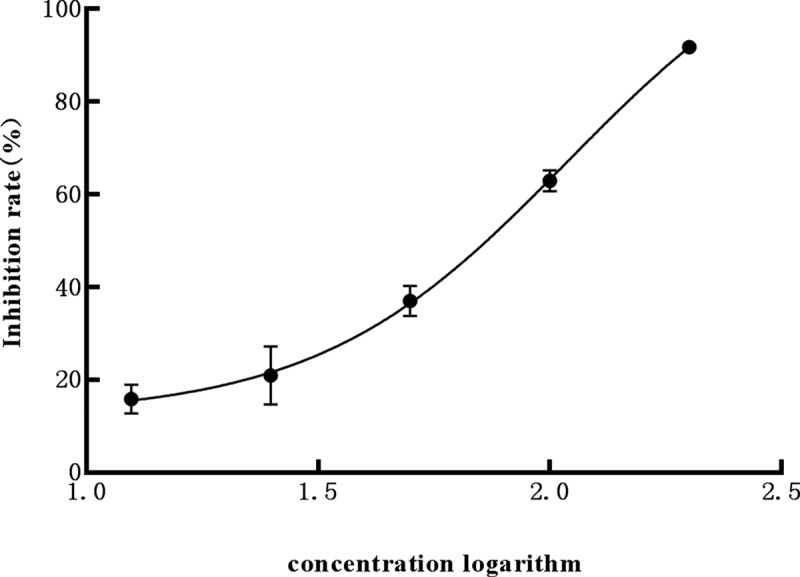
Inhibition rate of ethyl acetate extract with different concentration on *M. aeruginosa.*

The growth of algae was reflected by the absorption value at 680 nm of the hydroponic solution. The growth inhibition results indicated that the growth of *M. aeruginosa* can be affected by petroleum ether, dichloromethane, and ethyl acetate extracts at 200 mg L^−1^, and the inhibition rates reached 19.9%, 39.1% and 81.5%, respectively, on the 7th day. There was no significant difference between water phase and control at p < .05 (Figure 1). The ethyl acetate extract exhibited the strongest inhibited effect on *M. aeruginosa* during the test, with the EC_50_ of 59.6 mg L^−1^ on the 7th day (Figure 2;Table 1). Obviously, the ethyl acetate extract of *L. punctata* is regarded as the central polar part of algal inhibition activity on *M. aeruginosa*.

**Table 1.** Inhibition rate of four extracts on *M. aeruginosa* of different days

Extract	Time						
	1 d	2 d	3 d	4 d	5 d	6 d	7 d
Water phase	−5.18 ± 2.07a	−5.57 ± 1.08a	−10.82 ± 2.70a	10.75 ± 1.86a	8.96 ± 2.82a	4.61 ± 1.70a	4.79 ± 0.33a
Petroleum ether	−1.69 ± 2.63b	6.52 ± 0.07b	15.87 ± 2.55b	14.97 ± 0.56b	22.64 ± 1.97b	23.86 ± 1.56b	19.90 ± 1.69b
Dichloromethane	4.55 ± 2.57b	10.46 ± 0.33c	16.06 ± 1.80b	22.19 ± 1.31c	30.68 ± 2.80c	35.02 ± 1.69c	39.3 ± 2.52c
Ethyl acetale	15.23 ± 1.34b	25.22 ± 1.33d	43.68 ± 1.10c	60.45 ± 1.35d	70.43 ± 1.30d	77.37 ± 0.86d	81.57 ± 0.78d

Note: Data are presented as the mean ± S.D. (n = 3). Different letters in the same column indicate a significant difference at a level of 0.05 under 200 mg L^−1^.

### 3.2 *Content of Chlorophyll a on* M. aeruginosa

**Figure 3.**
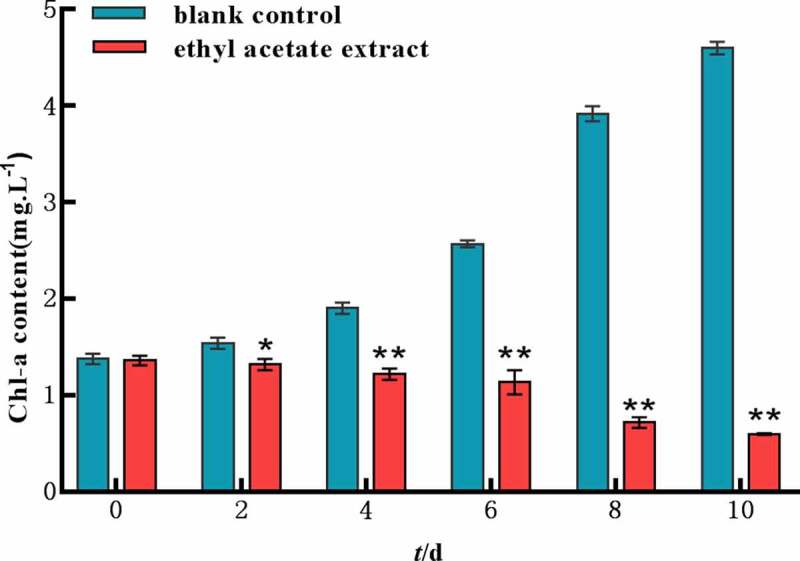
Chlorophyll a content of ethyl acetate extract on *M. aeruginosa.*

The results showed that the ethyl acetate extract has an inhibitory effect on Chl a level in cell pellets compared with the control at 200 mg L^−1^(Figure 3). In control group, the content of Chl a elevated with cell growth along test. On the 2th day, there was a significant difference between the experiment and control group. On the 10th day, it was 7.71 times the content of chlorophyll a in algae cells of experiment group which was only 0.598 mg L^−1^. Therefore, the ethyl acetate extract may interfere with the photosynthetic system of *M. aeruginosa*, which leads to a decrease of Chl a.

Error bars are one standard deviation of the mean (N = 3). Means that significantly differ from the controls are indicated with anasterisk*(oneway ANOVA; p < .05) or two asterisks**(p < .01).

### 3.3 *Contents of phycobiliproteins on* M. aeruginosa

**Figure 4.**
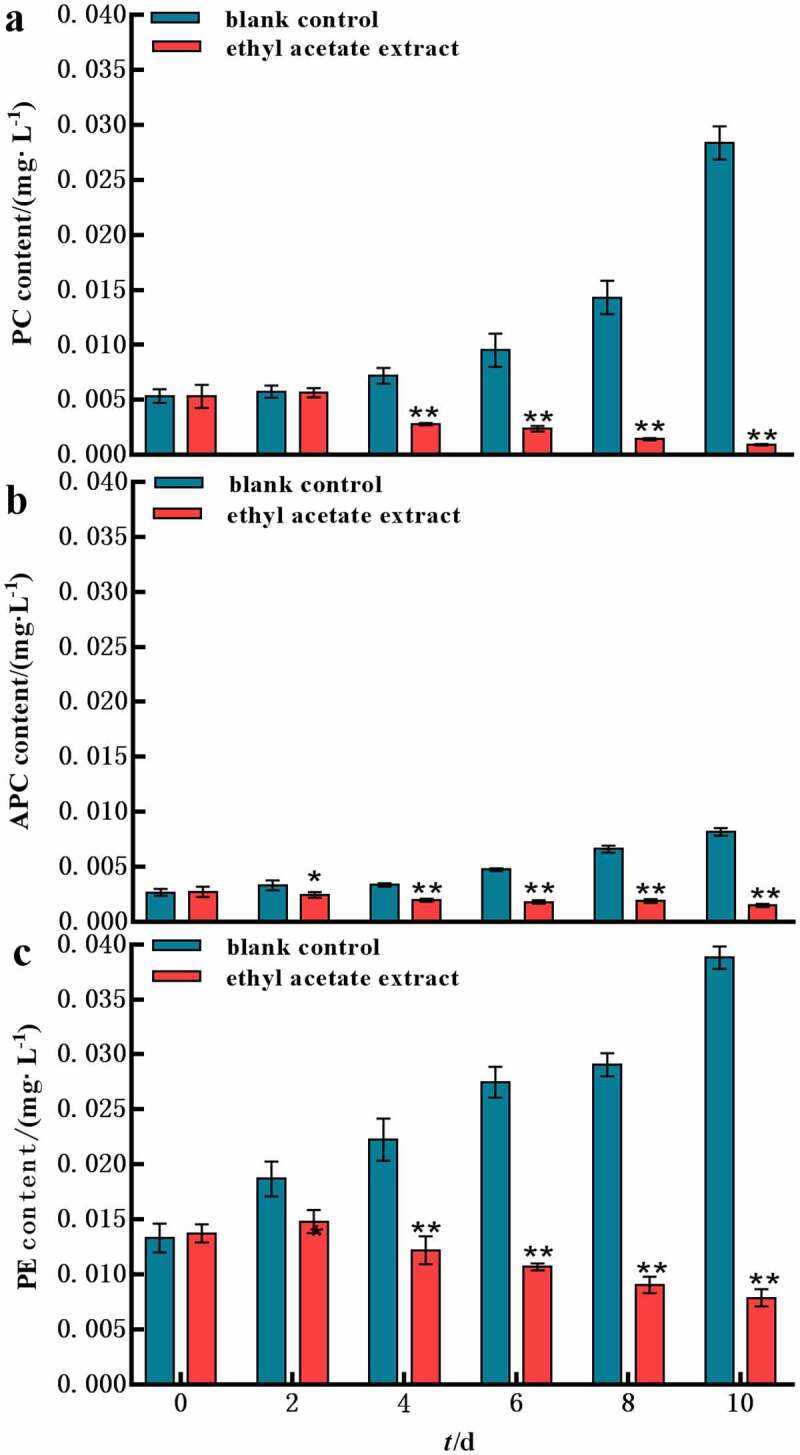
Phycobiliproteins content of ethyl acetate extract on *M. aeruginosa.*

The content of PBPs decreased gradually with the addition of 200 mg L^−1^ of ethyl acetate extract compared control group (Figure 4). The PBPs synthesis was strongest inhibited on the 4th day. Then the content of PBPs was lower than initial value. On the 10th day, the PBPs (PC, APC, PE) of experiment group decreased 19.9%, 39.1% and 81.5%, respectively. It manifested that the structure of PBPs was damaged by adding ethyl acetate extract of *L. punctata* and PC was susceptible. The results indicated that ethyl acetate extract could inhibit PBPs synthesis.

Error bars are one standard deviation of the mean (N = 3). Means that significantly differ from the controls are indicated with anasterisk*(onewayANOVA; p < .05) or two asterisks**(p < .01). a) the content of PC; b) the the content of APC; c) the content of PE.

### 3.4 *SOD activity in* M. aeruginosa

**Figure 5.**
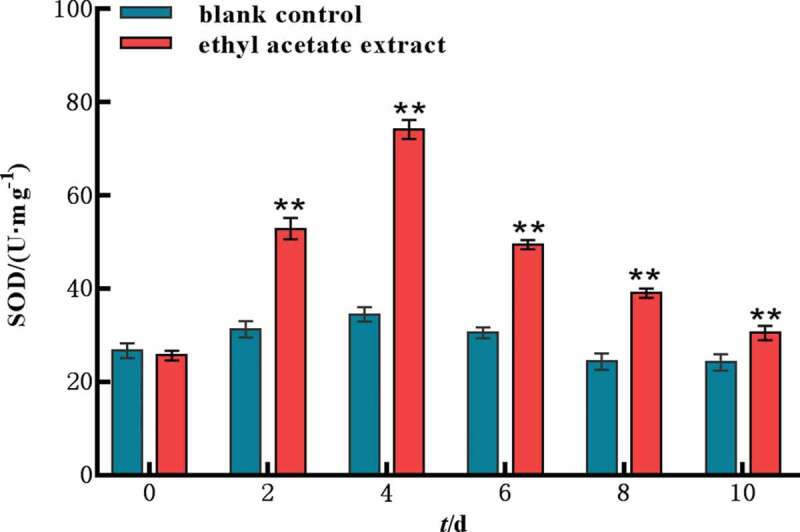
SOD activity of ethyl acetate extract on *M. aeruginosa.*

After the ethyl acetate extract was added, the SOD activity increased rapidly for the first four days, ranging from 25.67 U mg^−1^ to 74.13 U mg^−1^ (Figure 5). On the 4th day, the SOD activity was 2.15 times of that in the control group and then decreased next. These results indicated that the ethyl acetate extract had oxidative stress on *M. aeruginosa*, the SOD activity was increased to remove active oxygen and other harmful substances. SOD activity was decreased, because of the death of a large number of cell pellets.

Error bars are one standard deviation of the mean (N = 3). Means that significantly differ from the controls are indicated with anasterisk*(oneway ANOVA; p < .05) or two asterisks**(p < .01).

### 3.5 *MDA content in* M. aeruginosa

**Figure 6.**
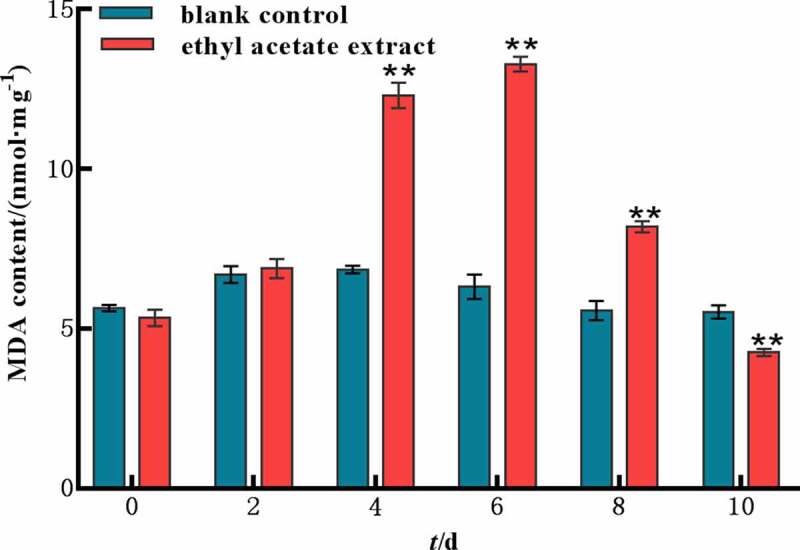
MDA content of ethyl acetate extract on *M. aeruginosa.*

As is shown in Figure 6, the content of MDA in the experimental group increased rapidly for the first six days and then decreased. The experimental group showed a significant stimulation effect with 200 mg L^−1^ of ethyl acetate extract. The content of MDA increased 1.84 times compared with that in the control group on the 4th day. The results indicated that ethyl acetate extract caused the overproduction of MDA.

Error bars are one standard deviation of the mean (N = 3). Means that significantly differ from the controls are indicated with anasterisk*(oneway ANOVA; p < .05) or two asterisks**(p < .01).

### 3.6 *H_2_O_2_ content in* M. aeruginosa

**Figure 7.**
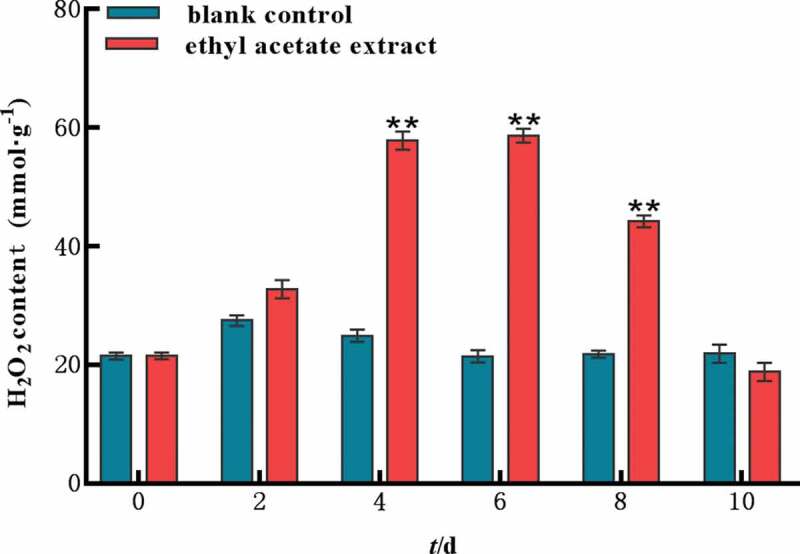
H_2_O_2_ content of ethyl acetate extract on *M.aeruginosa.*

The content of H_2_O_2_ in *M. aeruginosa* was increased remarkably and then decreased slowly. On the 6th day, the content of H_2_O_2_ reached maximum, with 58.7 mmol g^−1^, which was 2.7 folds of control (Figure 7). On the 10th day, the H_2_O_2_ in treatment groups was lower than control. The results indicated that the ethyl acetate extract exerted stress on the cells of *M*. aeruginosa, which enhanced the active oxygen metabolism in the cells and resulted in the accumulation of H_2_O_2_.

Error bars are one standard deviation of the mean (N = 3). Means that significantly differ from the controls are indicated with anasterisk*(oneway ANOVA; p < .05) or two asterisks**(p < .01).

### 3.7 *Effect of microcystin LR in* M. aeruginosa

**Figure 8.**
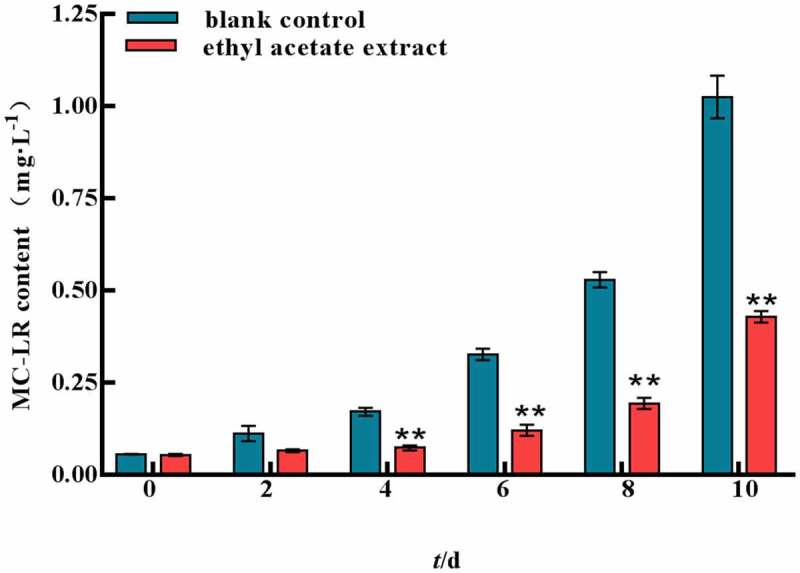
MC-LR content of ethyl acetate extract on *M.aeruginosa.*

Microcystins are secondary metabolites of *M. aeruginosa*. The changes of algal toxins are shown in Figure 8. With the extension of time, the ethyl acetate extract showed stronger inhibitory effects on MC-LR compared with the control group. On the 4th day, there was significant difference between control group and experimental group. For the first 6 days, the concentration of MC-LR in the experimental group increased slowly. The MC inhibition was 41.4% on the 10th day.

Error bars are one standard deviation of the mean (N = 3). Means that significantly differ from the controls are indicated with anasterisk*(oneway ANOVA; p < .05) or two asterisks**(p < .01).

## 4 Discussion

It was observed that submerged plant extracts including phenolic acids, terpenoids and alkaloids have strong cyanobacteria-suppressing functions and might be used directly to control the growth of cyanobacteria.^24^ Research using aquatic plants and their allelopathic effects in *M. aeruginosa* control has been extensively carried on in recent years.^25^ Duckweeds contain a variety of secondary metabolites, including phenolic compounds (favonoids, phenylpropanoids, and tannins) and terpenoids.^26^ *L. punctata* is known to have a higher favonoid content, such as apigenin, luteolin, vitexin, orientin and its derivatives, which have a variety of bioativities.^27,28^ Among them, the compound luteolin has been identified to inhibit the growth of M. aeruginosa remarkably with IC_50_ 6.5 mg/L.^21^ The favonoids compounds could be extracted by ethyl acetate which had significant algal inhibition activity in this study.

Allelochemicals induced damages on multiple levels of microalgal cells, including interfering the photosynthesis, generating oxidative stress, triggering programmed cell death, and disturbing other physiological and biochemical processes.^29^ Chl a is the most primary photosynthetic pigment in *M. aeruginosa* which is an indispensable index in photosynthetic rate and plays an essential part in energy capture and transfer during photosynthesis.^6^ PBPs can transfer absorbed energy to the photosynthetic system II of algae. These three PBPs (PC, APC, PE) are the main functional groups of cyanobacteria photosynthesis.^22^ In our experiment, the contents of chlorophyll a and PBPs were decreased indicated that the photosynthesis of algae was hindered. The results demonstrated that the chlorophyll a and PBPs may be the action sites of allelochemicals, resulting in failure of light energy capture or transmission, affecting the normal progress of photosynthesis, and thus significantly inhibiting the growth of cell pellets.

Environmental stress can disturb cell homeostasis and increase the production of reactive oxygen species. The antioxidant enzyme system is one of the important defense systems in plant response to stress, which can effectively protect plants and reduce the damage caused by environmental stress. The antioxidase SOD can catalyze the disproportionation of superoxide anion and generate hydrogen peroxide and molecular oxygen.^21^ MDA is the product of cellular lipid peroxidation, typically utilized as a marker of cellular physiological stress and oxidative damage.^22^ In this study, SOD, MDA and H_2_O_2_ showed a trend of increasing first and then decreasing by ethyl acetate extract, indicating a large number of reactive oxygen species produced in vivo and the cell membrane was damaged. However, cell pellets were irreversibly damaged under the long-term stress of ethyl acetate extract, resulting in a large number of death or decomposition and the contents of SOD, MDA and H_2_O_2_ in *M. aeruginosa* were decreased in the later stage. The MC yields of the ethyl acetate extract was significantly lower than those of the control group. This finding indicated that the ethyl acetate extract inhibited the synthesis of algal toxins indirectly while inhibiting the growth of *M. aeruginosa*.

In summary, this study finds that the ethyl acetate extract of *L. punctata* has significant inhibitory activity on the cell proliferation of *M. aeruginosa*. And the allelochemicals are concentrated in ethyl acetate extract which can affect the cyanobacterial cell membrane, photosynthetic system, PBPs synthesis, and intracellular MC-LR. However, it should be noted that the result of this research is limited and more detailed works are needed in further studies, such as other mechanisms besides lipid peroxidation and oxidative damage and the allelochemicals. Our results indicate that extracts of *L. punctata* is useful for controlling cyanobacterial blooms.
